# Proteomic data set of the organohalide-respiring Epsilonproteobacterium *Sulfurospirillum multivorans* adapted to tetrachloroethene and other energy substrates

**DOI:** 10.1016/j.dib.2016.06.022

**Published:** 2016-06-21

**Authors:** Tobias Goris, Christian L. Schiffmann, Jennifer Gadkari, Lorenz Adrian, Martin von Bergen, Gabriele Diekert, Nico Jehmlich

**Affiliations:** a Department of Applied and Ecological Microbiology, Institute of Microbiology, Friedrich Schiller University, 07743 Jena, Germany; b Department of Molecular Systems Biology, Helmholtz Centre for Environmental Research – UFZ, Permoserstrasse 15, 04318 Leipzig, Germany; c Department of Isotope Biogeochemistry, Helmholtz Centre for Environmental Research – UFZ, Permoserstrasse 15, 04318 Leipzig, Germany; d Institute of Biochemistry, Faculty of Biosciences, Pharmacy and Psychology, University of Leipzig, Talstrasse 33, 04103 Leipzig, Germany; e Department of Chemistry and Bioscience, Aalborg University, Fredrik Bajers Vej 7H, 9220 Aalborg East, Denmark

**Keywords:** Anaerobic respiration, Epsilonproteobacteria, Nitrate respiration, Reductive dechlorination, Reductive dehalogenase

## Abstract

*Sulfurospirillum multivorans* is a free-living, physiologically versatile Epsilonproteobacterium able to couple the reductive dehalogenation of chlorinated and brominated ethenes to growth (organohalide respiration). We present proteomic data of *S. multivorans* grown with different electron donors (formate or pyruvate) and electron acceptors (fumarate, nitrate, or tetrachloroethene [PCE]). To obtain information on the cellular localization of proteins, membrane extracts and soluble fractions were separated before data collection from both fractions. The proteome analysis of *S. multivorans* was performed by mass spectrometry (nanoLC-MS/MS). Raw data have been deposited at ProteomeXchange, “*ProteomeXchange provides globally coordinated proteomics data submission and dissemination*” [1], via the PRIDE partner repository with the dataset identifier PRIDE: PXD004011. The data might support further research in organohalide respiration and in the general metabolism of free-living Epsilonproteobacteria. The dataset is associated with a previously published study “Proteomics of the organohalide-respiring Epsilonproteobacterium *S. multivorans* adapted to tetrachloroethene and other energy substrates” [2].

**Specifications Table**

**Table:** 

Subject area	Microbiology
More specific subject area	Proteomics
Type of data	Table
How data was acquired	Ultimate 3000 RSLC (Thermo Scientific Dionex) coupled with a TriVersa NanoMate (Advion Biosciences, Norwich, UK) to an Orbitrap Fusion mass spectrometer (Thermo Scientific).
Data format	Raw
Experimental factors	Prior LC-MS/MS measurement
Experimental features	1) Cultivation of bacteria 2) Protein extraction 3) LC-MS/MS analysis
Data source location	Leipzig, Saxony, Germany
Data accessibility	Data are within this article. The mass spectrometry proteomics data have been deposited at ProteomeXchange via the PRIDE partner repository with the dataset identifier PRIDE: PXD004011.

## **Value of the data**

• Data set can be used for the characterization of organohalide respiration in Epsilonproteobacteria and to elucidate the electron transport chains between the electron donors formate or pyruvate and the electron acceptors fumarate, nitrate, or tetrachloroethene as well as the general stress response to chlorinated ethenes.
• The wide range of anaerobic substrates used in this study helps to get insights into the regulation of anaerobic respiration with emphasis on organohalide respiration in Epsilonproteobacteria.
• LC-MS/MS data provides a comprehensive proteome of *S. multivorans* including the information about the abundance of proteins in different nutritional conditions and can help to unravel anaerobic metabolism of free-living Epsilonproteobacteria.
• Separated analysis of the soluble and membrane fraction can deliver valuable information about cellular localization of closely related proteins in other Epsilonproteobacteria.

## 1. Data

In this dataset, we present the proteome of the organohalide-respiring, free-living Epsilonproteobacterium *Sulfurospirillum multivorans* grown with different electron donors (pyruvate or formate) and electron acceptors (fumarate, nitrate, or tetrachloroethene [PCE]). The data presented here are uploaded to PRIDE and contains i) LC-MS/MS data (^*^.raw) and ii) database search files (*.msf) of 36 LC-MS/MS measurements (three replicates of each, soluble fraction and membrane extract) of *S. multivorans* cultivated under 6 different anaerobic conditions (pyruvate with PCE, nitrate, or fumarate; formate with PCE, nitrate, or fumarate). The provided data are summarized in Table 1.

## 2 Experimental design, materials and methods

Here, we investigated the soluble/membrane proteome of *S. multivorans* in response to different energy substrate with different substrate combinations. Pyruvate/fumarate (Py/Fu) was used as the "standard" growth condition, the results of which were compared to the following electron donor/acceptor combinations: pyruvate with nitrate (Py/Ni) or PCE (Py/PCE); formate with fumarate (Fo/Fu), PCE (Fo/PCE) or nitrate (Fo/Ni). We explored the downregulation of the organohalide respiratory machinery in *S. multivorans* when the cells were cultivated for a long time (about 100 generations) in the absence of chlorinated ethenes [3]. For the generation of these “organohalide-respiration-silent” cells, pyruvate/fumarate was used as substrate combination for 60 transfers. The whole set was prepared for LC-MS/MS analysis and the acquired data are provided.

## 3 Growth of Sulfurospirillum multivorans

The defined mineral medium used for growth of *S. multivorans* (German Collection of Microorganisms [DSMZ] number 12446) was prepared as previously described [4]. Oxygen was removed with 30 cycles gas evacuation and flushing with molecular nitrogen. The following modification to the original medium was used: The medium was prepared without cyanocobalamin (vitamin B_12_). The cultivation of *S. multivorans* was performed at 28 °C under continuous shaking in rubber-stoppered 2 L glass flasks (Schott, Germany). Precultures for inoculation of the main cultures were grown in rubber stoppered 200 mL serum bottles; transfers were always performed with 10% culture volume. All precultures were inoculated with cultures grown with pyruvate and fumarate for 60 transfer steps to generate cells lacking proteins involved in organohalide respiration [3]. Before inoculation of the main culture, the precultures were transferred three consecutive times into the desired medium with a 10% inoculum in order to avoid transfer of pyruvate or fumarate from the initial preculture. The ratio between medium and gas phase (v/v) during all growth experiments was 1 to 1. The electron donors pyruvate and formate and the electron acceptors fumarate and nitrate were used at a concentration of 40 mM, each, except in the combination of formate and nitrate, where nitrate was supplied at a concentration of 10 mM. PCE was dissolved in hexadecane (0.5 M stock solution). Concentration of PCE in the medium was 10 mM. Acetate (5 mM) was added as carbon source when formate was used as electron donor and those media were supplemented with 0.05% yeast extract. Titanium(III)-citrate (5.6 µM) was added when *S. multivorans* was grown with formate and nitrate. Overall, six electron donor/acceptor conditions were used: the standard condition pyruvate/fumarate (Py/Fu) and five conditions for comparison: pyruvate/nitrate (Py/Ni), pyruvate/PCE (Py/PCE), formate/fumarate (Fo/Fu), formate/nitrate (Fo/Ni) and formate/PCE (Fo/PCE).

## 4 Cell harvesting and sample preparation

Cells of each growth condition were grown in 3×1 L medium to the late exponential growth phase and then harvested via centrifugation at 12,000 g for 10 min at 10 °C. The cell pellets were washed and resuspended in a ratio of 1:2 in 50 mM Tris HCl buffer (pH 7.5). One tip of a spatula DNase I (AppliChem, Darmstadt, Germany) and one tablet protease inhibitor (cOmplete Mini, EDTA-free; Roche, Mannheim, Germany) per 10 mL buffer were added. Cell disruption was performed with a French Press at 1000 psi (6.89 MPa) followed by removal of cell debris by centrifugation at 6000*g* for 10 min at 4 °C. The resulting supernatant was ultracentrifuged (260,000x*g* for 45 min at 4 °C) and the resulting soluble protein fraction (SF) in the supernatant was carefully decanted. The residual pellet (membrane fraction) was washed twice with protease inhibitor amended washing buffer (see above) and afterwards resuspended in solubilization buffer (300 mM NaCl, 100 mM Tris–HCl, pH 7.5) containing 1% (w/v) digitonin (AppliChem, Darmstadt, Germany) as detergent. This mixture was stirred overnight at 16 °C and subsequently ultracentrifuged (260,000x*g*, for 45 min at 4 °C). The resulting supernatants are designated as membrane protein extracts (ME).

## 5 Proteomics by short SDS-PAGE

We determined the protein amount using the Bio-Rad Bradford assay (Bio-Rad, Munich, Germany). 50 µg protein lysates were applied to run shortly (1.5 cm) on a 12% SDS-PAGE (1.0 mm thickness, running condition: 10 mA for 10 min followed by 20 mA for 20 min). Each sample lane was cut into four pieces of about 5 mm each, which were prepared for proteolytic cleavage by reduction and alkylation separately. Protein digestion was carried out with sequencing-grade trypsin (Promega, Madison, WI, USA). The resulting peptides were desalted and extracted with C_18_ ZipTips (Merck Millipore, Darmstadt, Germany). The four peptide lysates from one sample lane were unified before LC-MS/MS analysis.

## 6 Mass spectrometry and data analysis

Mass spectrometric analysis was performed by separation of tryptic peptides using an Ultimate 3000 nanoRSLC (Thermo Scientific, Germany) system coupled to an Orbitrap Fusion mass spectrometer (Thermo Scientific, San Jose, CA, USA). We used two mobile phases: mobile phase A was 0.1% formic acid, mobile phase B contained 80% acetonitrile and 0.08% formic acid. Peptides were loaded for 5 min on the precolumn (µ-precolumn, cartridge column, 3 µm particle size, 75 µm inner diameter, 2 cm, particles C_18_, Thermo Scientific) at 4% mobile phase B and eluted from the analytical column (PepMap Acclaim C_18_ LC Column, 15 cm, 3 µm particle size, Thermo Scientific) over a 90 min gradient of mobile phase B (4-55% B). The MS setting were described in detail [2].

The software Proteome Discoverer (v1.4.1.14, Thermo Scientific) was used for protein identification and the acquired MS/MS spectra were searched with the Sequest HT and MS Amanda algorithms against the *S. multivorans* genome database with 3191 non-redundant CDS (as of February, 17th 2014 available from the NCBI Genbank database, accession number CP007201, [5]). Settings of Sequest HT and MS Amanda were as follows: trypsin digestion, dynamic oxidation of methionine and carbamidomethylation of cysteine as fixed. Up to two missed cleavages were allowed, MS mass tolerance was set to 10 ppm and the MS/MS mass tolerance to 0.05 Da. The quantitative information of the proteins was calculated by the average of the top three peptides per protein with the precursor ion area detector implemented in the Proteome Discoverer. The database search result files (*.msf) are also uploaded to PRIDE and can be accessed.

## Figures and Tables

**Table 1:** *Sulfurospirillum multivorans* proteome data that are provided at PRIDE. For each condition three biological samples were processed.

**Cultivation condition**			**Sample name for LC-MS/MS and database search file** (numbers indicates the replicates)
**Electron donor**	**Electron acceptor**	**Extract/Fraction**	
Pyruvate (Py)	tetrachloroethene (PCE)	Soluble	*_Smult_PyPCELE1
			*_Smult_PyPCELE2
			*_Smult_PyPCELE3
		Membrane	*_Smult_PyPCEME1
			*_Smult_PyPCEME2
			*_Smult_PyPCEME3
			
Pyruvate (Py)	nitrate (Ni)	Soluble	*_Smult_PyNOLE1
			*_Smult_PyNOLE2
			*_Smult_PyNOLE3
		Membrane	*_Smult_PyNOME1
			*_Smult_PyNOME2
			*_Smult_PyNOME3
			
Pyruvate (Py)	fumarate (Fu)	Soluble	*_Smult_PyFuLE1
			*_Smult_PyFuLE2
			*_Smult_PyFuLE3
		Membrane	*_Smult_PyFuME1
			*_Smult_PyFuME2
			*_Smult_PyFuME3
			
			
Formate (Fo)	tetrachloroethene (PCE)	Soluble	*_Smult_FoPCELE1
			*_Smult_FoPCELE2
			*_Smult_FoPCELE3
		Membrane	*_Smult_FoPCEME1
			*_Smult_FoPCEME2
			*_Smult_FoPCEME3
			
Formate (Fo)	nitrate (Ni)	Soluble	*_Smult_FoNOLE1
			*_Smult_FoNOLE2
			*_Smult_FoNOLE3
		Membrane	*_Smult_FoNOME1
			*_Smult_FoNOME2
			*_Smult_FoNOME3
			
Formate (Fo)	fumarate (Fu)	Soluble	*_Smult_FoFuLE1
			*_Smult_FoFuLE2
			*_Smult_FoFuLE3
		Membrane	*_Smult_FoFuME1
			*_Smult_FoFuME2
			*_Smult_FoFuME3
